# High Prevalence of Pseudo-Pelger–Huët Anomaly and Neutrophil Dysplasia in Children with Celiac Disease: A Retrospective Case–Control Study

**DOI:** 10.3390/children13091158

**Published:** 2026-08-28

**Authors:** Burçak Kurucu, Gülseren Şahin

**Affiliations:** 1 Department of Pediatric Hematology and Oncology, Ankara Etlik City Hospital, 06170 Ankara, Türkiye; 2 Department of Pediatric Gastroenterology and Hepatology, Ankara Etlik City Hospital, 06170 Ankara, Türkiye

**Keywords:** celiac disease, Pseudo-Pelger–Huët anomaly, neutrophil dysplasia, peripheral blood smear, extraintestinal manifestations, systemic inflammation

## Abstract

**Highlights:**

**What are the main findings?**

• Children with celiac disease demonstrate a significantly high prevalence of pseudo–Pelger–Huët anomaly (87.1% vs. 8.1%) and neutrophil dysplasia (52.9% vs. 1.4%) compared to healthy controls.
• Multivariable logistic regression demonstrates that celiac disease is independently associated with an 81-fold higher odds of pseudo–Pelger–Huët anomaly and a 94-fold higher odds of neutrophil dysplasia.

**What are the implications of the main finding?**

• Peripheral blood smear evaluation shows significant diagnostic differences when comparing childhood celiac disease patients to healthy controls (AUC = 0.924, sensitivity = 90.0%, specificity = 91.9%).
• Recognizing these benign, non-malignant morphologic neutrophil abnormalities as systemic features of celiac disease can prevent unnecessary invasive hematological workups, such as bone marrow aspiration.

**Abstract:**

**Background/Objectives:** Celiac disease (CD) is a chronic immune-mediated disorder associated with a broad spectrum of extraintestinal manifestations, including hematological abnormalities. Although anemia and micronutrient deficiencies are well recognized, neutrophil morphological abnormalities have not been systematically investigated in pediatric CD; this study aimed to evaluate peripheral blood smear findings in children with CD compared with healthy controls. **Methods:** This retrospective case–control study included 70 children with CD and 74 healthy controls; demographic characteristics, anthropometric measurements, hematological and biochemical parameters, and peripheral blood smear findings were analyzed and compared between groups. **Results:** Children with CD had significantly higher frequencies of underweight status (15.7% vs. 2.7%) and short stature (21.4% vs. 1.4%) than controls. Most hematological and biochemical parameters were comparable between groups, except for platelet counts and ferritin levels, which differed significantly. Pseudo-Pelger–Huët anomaly (PPHA) was identified in 87.1% of children with CD versus 8.1% of controls (*p* < 0.001), and neutrophil dysplasia was observed in 52.9% versus 1.4%, respectively (*p* < 0.001). After adjustment for age and sex, CD remained strongly associated with PPHA (adjusted OR 81.19, 95% CI 28.65–278.45), neutrophil dysplasia (adjusted OR 93.85, 95% CI 18.46–1724.96), and the combined presence of both abnormalities (adjusted OR 79.89, 95% CI 15.86–1461.89) (all *p* < 0.001). ROC analysis demonstrated excellent discrimination between CD and controls (AUC = 0.924; sensitivity = 90.0%; specificity = 91.9%). Within the CD cohort, PPHA and dysplasia were not associated with celiac antibody positivity, gluten-free diet adherence, vitamin D deficiency, or comorbid conditions. **Conclusions:** To our knowledge, this is the first study to systematically evaluate peripheral blood smear morphology in pediatric CD, demonstrating a remarkably high prevalence of PPHA and neutrophil dysplasia. These findings suggest that neutrophil morphological abnormalities may represent an underrecognized hematological manifestation of pediatric CD, potentially reflecting chronic immune-mediated alterations in granulopoiesis. Further prospective studies are needed to clarify their pathogenesis, clinical significance, and reversibility following long-term gluten-free diet treatment.

## 1. Introduction

Celiac disease (CD) is a chronic immune-mediated enteropathy triggered by dietary gluten in genetically predisposed individuals, particularly those carrying HLA-DQ2 and/or HLA-DQ8 haplotypes [1]. It is now recognized as a multisystem disease involving small-bowel mucosal injury, disease-specific antibodies, epithelial stress responses, and cytokine-mediated inflammation, in which interleukin-15 and interferon-γ play central roles [2,3,4,5]. CD affects approximately 1% of the global population, with its reported prevalence increasing because of improved diagnostic approaches and greater clinical awareness [6,7]. In children, its presentation is heterogeneous and may include gastrointestinal symptoms, growth impairment, and hematological, endocrinological, neurological, dermatological, or rheumatological manifestations [1,8]. Because extraintestinal findings may precede or overshadow gastrointestinal symptoms, their recognition is important for early diagnosis and appropriate long-term follow-up [9,10]. Among the extraintestinal manifestations of CD, hematologic abnormalities are particularly common. Anemia remains the most frequently reported finding and is often multifactorial, resulting from iron, folate, or vitamin B12 deficiency, chronic inflammation, or combinations thereof. In addition, thrombocytopenia, thrombocytosis, leukocyte abnormalities, coagulation disorders, thromboembolic events, hyposplenism, immunoglobulin A deficiency, and, rarely, lymphoma have been described [11,12,13,14,15]. Nutritional studies further suggest that deficiencies of iron, folate, vitamin B12, and other micronutrients may persist around the time of diagnosis, creating conditions that could affect normal hematopoiesis and myeloid maturation [16,17].

Neutrophils are essential components of innate immunity, and their segmented nuclei facilitate cellular deformability and migration. Abnormalities in nuclear segmentation or cytoplasmic granulation may indicate disturbed granulopoiesis. Dysgranulopoiesis includes hyposegmentation, abnormal chromatin condensation, atypical nuclear forms, and cytoplasmic hypogranulation and may occur in both clonal and nonclonal conditions [18,19,20]. Pseudo-Pelger–Huët anomaly (PPHA) is one of the most recognizable forms of acquired neutrophil dysplasia. Morphologically, affected neutrophils demonstrate marked nuclear hyposegmentation with bilobed, pince-nez-like, or unilobed nuclei accompanied by coarse chromatin clumping. Although PPHA is classically associated with myelodysplastic syndromes and other myeloid neoplasms, it is not specific to these disorders. Acquired forms have also been reported in association with medications, infections, inflammatory conditions, autoimmune diseases, and nutritional deficiencies [20,21,22,23,24]. Recognition of this distinction is clinically important because reactive or reversible PPHA may be misinterpreted as evidence of primary myelodysplasia, potentially leading to unnecessary invasive investigations.

The possibility that children with CD may develop PPHA or related neutrophil dysplastic changes is biologically plausible. Malabsorption-related deficiencies of folate and vitamin B12 are established nonclonal causes of dysgranulopoiesis and may produce morphologic abnormalities that mimic myelodysplastic syndromes, including PPHA-like forms [17,25]. Furthermore, active CD is characterized by persistent immune activation involving IL-15, interferon-γ, and other inflammatory pathways that influence both adaptive and innate immune responses [3,5,26]. Although a direct causal relationship between CD and neutrophil dysplasia has not been established, the combined effects of chronic inflammation, immune dysregulation, and micronutrient deficiency may plausibly influence granulopoiesis and neutrophil maturation.

Despite the extensive literature describing anemia and other hematologic manifestations of CD, neutrophil morphology has received little attention. Existing studies primarily focus on anemia, thrombocytosis, thrombosis, hyposplenism, and lymphoproliferative complications, whereas reports addressing neutrophil dysplasia in CD are scarce [14,27]. For example, a pediatric case report described bone marrow suppression with neutropenia in a child with CD, with hematological recovery following initiation of a gluten-free diet [27]. However, that report focused on quantitative hematological abnormalities and did not provide a systematic assessment of neutrophil morphology. Similarly, the PPHA literature is dominated by myelodysplastic syndromes, drug-related changes, transplantation, and other non-celiac conditions [20,24]. This disparity suggests an important knowledge gap and raises the possibility that dysplastic neutrophil features may be underrecognized in children with CD because peripheral blood smear morphology is not routinely evaluated in this context.

Therefore, the present study aimed to evaluate hematologic findings, peripheral blood smear characteristics, and the prevalence of pseudo-Pelger–Huët anomalies and other neutrophil dysplastic features in children with celiac disease. We hypothesized that PPHA and neutrophil dysplasia would be more frequent in children with celiac disease than in healthy controls and might represent underrecognized hematological features of pediatric celiac disease. Demonstration of such an association would broaden the recognized hematologic spectrum of pediatric CD and help prevent misinterpretation of peripheral smear abnormalities as evidence of primary myelodysplasia.

## 2. Materials and Methods

### 2.1. Study Design and Setting

This retrospective case–control study was conducted at the Pediatric Hematology and Oncology Clinic and the Pediatric Gastroenterology and Hepatology Clinic of Health Sciences University Ankara Dr. Sami Ulus Maternity, Child Health and Diseases Training and Research Hospital. Medical records, laboratory findings, and archived peripheral blood smear preparations obtained during routine clinical follow-up between March 2021 and September 2021 were reviewed.

### 2.2. Study Population

A total of 144 children were included in the study, comprising 70 patients with confirmed celiac disease and 74 healthy controls. The diagnosis of celiac disease was established according to clinical findings, serological testing, and histopathological evaluation when indicated. Healthy controls were selected from children attending the pediatric hematology outpatient clinic for routine control evaluations. At our institution, peripheral blood smear examination is routinely performed as part of the clinical assessment of children presenting to this clinic. Therefore, the control smears were obtained during routine clinical care and were not prepared specifically for research purposes. Children were included as controls only when their clinical and laboratory evaluations showed no evidence of chronic inflammatory, autoimmune, hematological, infectious, or malignant disease. Patients with acute severe infection, known hematological disease, malignancy, immunodeficiency, or inadequate clinical or laboratory records were excluded. The study protocol was approved by the Institutional Ethics Committee (Approval No. E-21/03-117). As this was a retrospective study based solely on pre-existing medical records and archived laboratory specimens obtained during routine clinical care, the requirement for individual informed consent was waived by the Ethics Committee, and patient data were anonymized prior to analysis.

### 2.3. Clinical and Anthropometric Data Collection

Demographic and clinical data were retrieved from medical records, including age, sex, date of diagnosis, follow-up duration, celiac serology status, gluten-free diet adherence, presence of comorbid conditions, medication use, and infection history at the time of blood sampling. Height and weight measurements were evaluated according to age- and sex-specific Turkish reference growth charts. Underweight and short stature were defined as weight-for-age and height-for-age values below −2 standard deviations, respectively [10].

### 2.4. Laboratory Evaluations

Complete blood count parameters, including white blood cell count (WBC), red blood cell count (RBC), hemoglobin (HGB), hematocrit (HCT), mean corpuscular volume (MCV), red cell distribution width (RDW), and platelet count (PLT), were obtained from routine laboratory records. Biochemical parameters included serum concentrations of vitamin B12, folate, ferritin, and vitamin D. Vitamin B12 deficiency was defined as serum vitamin B12 < 200 pg/mL, ferritin deficiency as ferritin < 12 ng/mL, and vitamin D deficiency as serum 25-hydroxyvitamin D < 30 ng/mL. Celiac serological assessment included anti-tissue transglutaminase antibodies, anti-gliadin IgA/IgG antibodies, and anti-endomysial antibodies.

### 2.5. Peripheral Blood Smear Assessment

Archived peripheral blood smears stained according to routine laboratory procedures were examined using light microscopy. Differential leukocyte counts were performed by evaluating 100 consecutive leukocytes per smear. Neutrophils were assessed for the presence of pseudo-Pelger–Huët anomaly and dysplastic morphological changes. Pseudo-Pelger–Huët anomaly was defined by hyposegmented neutrophil nuclei with characteristic bilobed or unsegmented morphology. Dysplastic changes included abnormal nuclear morphology and maturation defects identified during microscopic evaluation. The percentage of neutrophils demonstrating pseudo-Pelger–Huët morphology was recorded for each patient. For the purposes of this study, PPHA positivity was operationally defined as the presence of PPHA morphology in ≥5% of the evaluated neutrophils. PPHA cells were identified by nuclear hyposegmentation, including unilobed or bilobed nuclei, accompanied by coarse, mature chromatin clumping. To minimize observer bias, all peripheral blood smears were coded and presented in a randomized order by the pediatric gastroenterologist. The pediatric hematologist who performed the morphological assessment was blinded to group allocation and did not know whether a smear belonged to a child with celiac disease or a control participant. Group allocation was revealed only after all morphological assessments were completed and recorded.

### 2.6. Statistical Analysis

All statistical analyses were performed using R version 4.5.1. The distribution of continuous variables was assessed using the Shapiro–Wilk test. Continuous variables were summarized as mean ± standard deviation (SD) or mean with 95% confidence interval (CI), as appropriate, whereas categorical variables were expressed as frequencies and percentages. Comparisons between the celiac disease and control groups were performed using Student’s *t*-test for continuous variables and Pearson’s chi-square test or Fisher’s exact test for categorical variables, as appropriate.

Because some peripheral blood smear abnormalities were observed in very few controls, crude and age- and sex-adjusted odds ratios (ORs) were estimated using Firth’s penalized logistic regression to reduce sparse-data bias and address potential quasi-complete separation. Separate models were fitted for PPHA, neutrophil dysplasia, and the simultaneous presence of both abnormalities. Odds ratios with 95% profile penalized-likelihood CIs were reported. Firth’s penalized logistic regression was performed using the logistf package, version 1.26.0.

For ROC analysis, a composite peripheral blood smear score was created based on the presence of PPHA and neutrophil dysplasia. The score ranged from 0 to 2: a score of 0 indicated the absence of both abnormalities, 1 indicated the presence of either PPHA or neutrophil dysplasia, and 2 indicated the presence of both abnormalities. This composite score alone was used as the predictor for distinguishing children with celiac disease from healthy controls; age and sex were not included in the ROC model. The optimal cutoff was determined using the Youden index, and the area under the ROC curve (AUC), sensitivity, and specificity were calculated. Correlations between peripheral blood smear findings and hematological and biochemical parameters were evaluated using Spearman’s rank correlation analysis. All statistical tests were two-sided, and a *p*-value < 0.05 was considered statistically significant.

## 3. Results

### 3.1. Demographic and Anthropometric Characteristics

A total of 144 children were included in the study: 70 with celiac disease and 74 healthy controls. The mean age was comparable between the two groups (9.79 ± 4.18 years in the celiac disease group vs. 8.69 ± 4.95 years in the control group, *p* > 0.05). Similarly, the proportion of female participants did not differ between groups (57.1% vs. 56.8%, *p* > 0.05), indicating a balanced distribution of age and sex. Anthropometric assessment revealed significant differences between the study groups. Underweight status, defined as weight-for-age below −2 standard deviations, was observed in 15.7% of children with celiac disease compared with 2.7% of controls (*p* < 0.05). Likewise, short stature, defined as height-for-age below −2 standard deviations, was substantially more frequent among children with celiac disease than among healthy controls (21.4% vs. 1.4%, *p* < 0.05). Demographic and anthropometric characteristics of the study groups are shown in Table 1.

### 3.2. Hematological and Biochemical Findings

The hematological and biochemical findings of children with celiac disease and healthy controls are presented in Table 2. Total white blood cell counts were comparable between the two groups, with mean values of 6881.9/µL (95% CI: 6488.1–7275.7) in the celiac disease group and 7104.2/µL (95% CI: 6636.9–7571.6) in the control group (*p* > 0.05). Similarly, no significant differences were observed in red blood cell count, hemoglobin concentration, hematocrit, or mean corpuscular volume between the groups (all *p* > 0.05). Red cell distribution width tended to be higher in children with celiac disease than in controls (13.8% vs. 13.2%), although this difference did not reach statistical significance. Vitamin B12, folate, and vitamin D concentrations were also similar between the two groups (all *p* > 0.05).

Among the evaluated laboratory parameters, platelet count and ferritin concentration differed significantly between groups. Children with celiac disease had higher platelet counts than healthy controls (334,851.4/µL [95% CI: 313,660.2–356,042.5] vs. 305,442.9/µL [95% CI: 287,185.2–323,700.5], *p* < 0.05). Ferritin levels were also significantly different between the groups, with mean values of 30.1 ng/mL (95% CI: 17.3–42.9) in children with celiac disease and 28.6 ng/mL (95% CI: 25.1–32.0) in controls (*p* < 0.05).

### 3.3. Peripheral Blood Smear Findings

The frequency of peripheral blood smear abnormalities differed markedly between children with celiac disease and healthy controls (Table 3). Pseudo-Pelger–Huët anomaly was identified in 87.1% of children with celiac disease, whereas only 8.1% of controls exhibited this finding (*p* < 0.001). Neutrophil dysplasia was also substantially more common in the celiac disease group. Dysplastic changes were observed in 52.9% of children with celiac disease compared with 1.4% of healthy controls (*p* < 0.001). The coexistence of the Pseudo-Pelger–Huët anomaly and neutrophil dysplasia was detected in half of the children with celiac disease (50.0%), while this combination was observed in only 1.4% of controls (*p* < 0.001).

**Figure 1:**
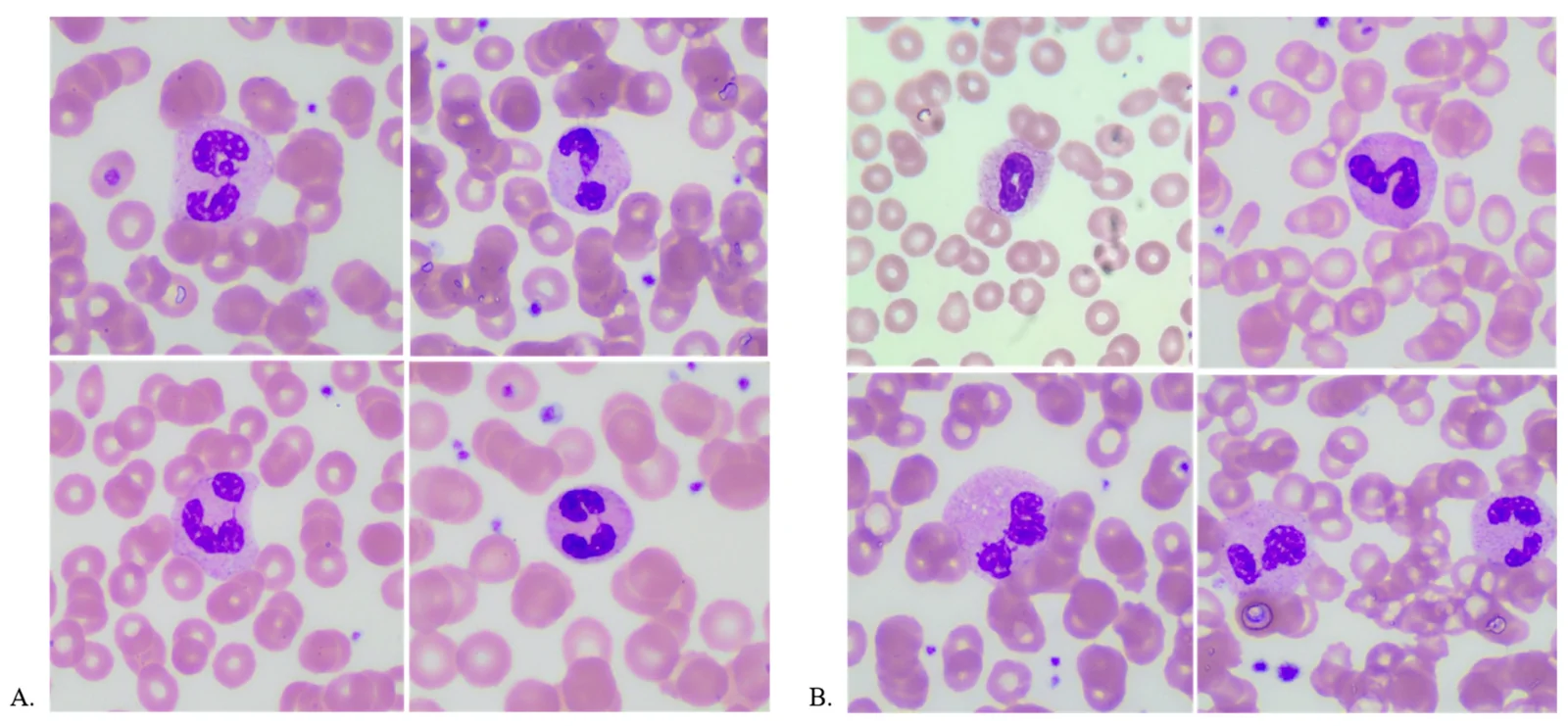
Representative peripheral blood smear findings in children with celiac disease. (**A**) Neutrophils demonstrating pseudo-Pelger–Huët anomaly, characterized by typical bilobed nuclear hyposegmentation with mature, clumped chromatin. (**B**) Dysplastic neutrophils exhibiting prominent cytoplasmic hypogranulation alongside abnormal nuclear morphology. Peripheral blood smears were evaluated by light microscopy during a 100-cell differential count (Wright–Giemsa stain, ×100 oil immersion).

Among the evaluated laboratory and morphological parameters, peripheral blood smear abnormalities showed the greatest differences between the study groups. In particular, the Pseudo-Pelger–Huët anomaly was present in nearly nine out of ten children with celiac disease, whereas dysplastic neutrophil changes affected approximately one-half of the patient cohort. Dysplastic and PHA-like neutrophils are shown in Figure 1. When analyzed as a continuous variable, the PPHA percentage was significantly higher in children with celiac disease than in controls (median [IQR]: 6.6% [3.8–10.4] vs. 0.0% [0.0–0.0], *p* < 0.001).

### 3.4. Clinical Relevance of Peripheral Blood Smear Abnormalities

#### 3.4.1. Multivariable Logistic Regression Analysis

To determine whether the observed peripheral blood smear abnormalities were independently associated with celiac disease, multivariable logistic regression analyses adjusted for age and sex were performed (Table 4). After adjustment, celiac disease remained strongly associated with all evaluated smear abnormalities.

Children with celiac disease had an 81-fold higher likelihood of exhibiting Pseudo-Pelger–Huët anomaly compared with healthy controls (adjusted OR: 81.19, 95% CI: 28.65–278.45, *p* < 0.001). Similarly, the odds of neutrophil dysplasia were nearly 94 times higher in children with celiac disease than in controls (adjusted OR: 93.85, 95% CI: 18.46–1724.96, *p* < 0.001). The coexistence of Pseudo-Pelger–Huët anomaly and neutrophil dysplasia was also strongly associated with celiac disease, with affected children demonstrating approximately 80-fold higher odds of presenting both abnormalities simultaneously (adjusted OR: 79.89, 95% CI: 15.86–1461.89, *p* < 0.001). Adjustment for age and sex had minimal impact on the magnitude of these associations, indicating that the relationship between celiac disease and peripheral blood smear abnormalities was largely independent of demographic characteristics.

#### 3.4.2. Clinical Correlates Within the Celiac Disease Group

**Figure 2:**
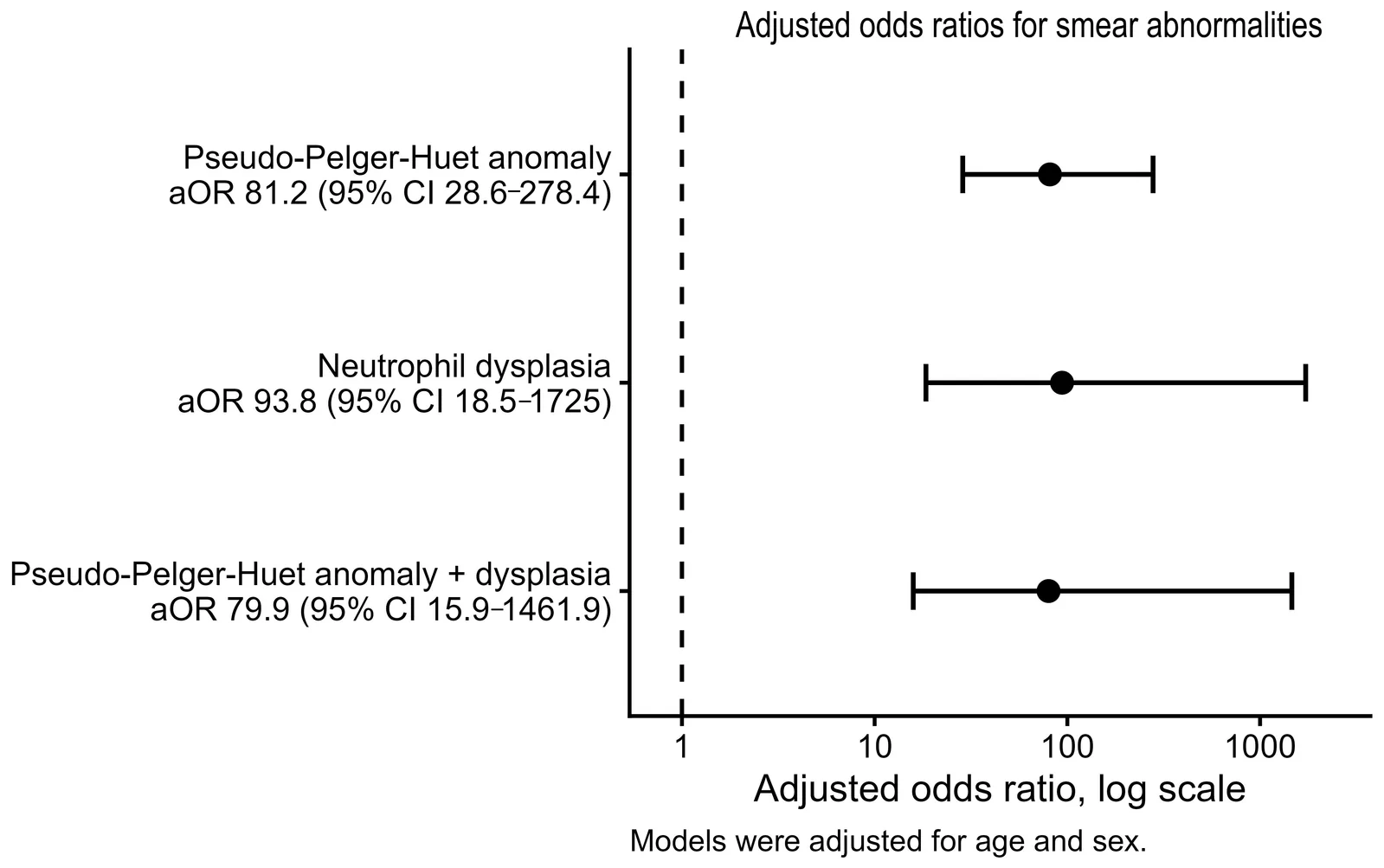
Forest plot showing adjusted odds ratios (aORs) and 95% confidence intervals for peripheral blood smear abnormalities in children with celiac disease compared with healthy controls. Estimates were derived from multivariable logistic regression models adjusted for age and sex. All evaluated smear abnormalities remained strongly associated with celiac disease after adjustment.

Associations between peripheral blood smear abnormalities and selected clinical characteristics of children with celiac disease are presented in Table 5. The prevalence of the Pseudo-Pelger–Huët anomaly did not differ according to celiac antibody positivity, adherence to a gluten-free diet, presence of comorbid conditions, or vitamin D deficiency (all *p* > 0.05). Similarly, no significant associations were observed between neutrophil dysplasia and any of the evaluated clinical variables (all *p* > 0.05). Notably, both Pseudo-Pelger–Huët anomaly and neutrophil dysplasia were observed across a broad range of patients, irrespective of serological status or dietary adherence. The absence of significant associations suggests that these morphological abnormalities are not restricted to specific clinical subgroups of children with celiac disease. Instead, they appear to represent a common hematological feature within the celiac cohort examined in the present study. Although the frequencies of smear abnormalities varied numerically across some subgroups, none of these differences were statistically significant. Figure 2 summarizes the age- and sex-adjusted associations between celiac disease and the evaluated peripheral blood smear abnormalities. The strongest association was observed for neutrophil dysplasia (adjusted OR: 93.85, 95% CI: 18.46–1724.96), followed by PPHA (adjusted OR: 81.19, 95% CI: 28.65–278.45) and the combined presence of PPHA and dysplasia (adjusted OR: 79.89, 95% CI: 15.86–1461.89). Despite adjustment for age and sex, all smear abnormalities remained strongly associated with celiac disease, with confidence intervals consistently excluding the null value.

Although no statistically significant differences were identified, several subgroups contained relatively few participants. Therefore, these exploratory findings should be interpreted cautiously and should not be considered evidence of the absence of clinically relevant associations.

**Figure 3:**
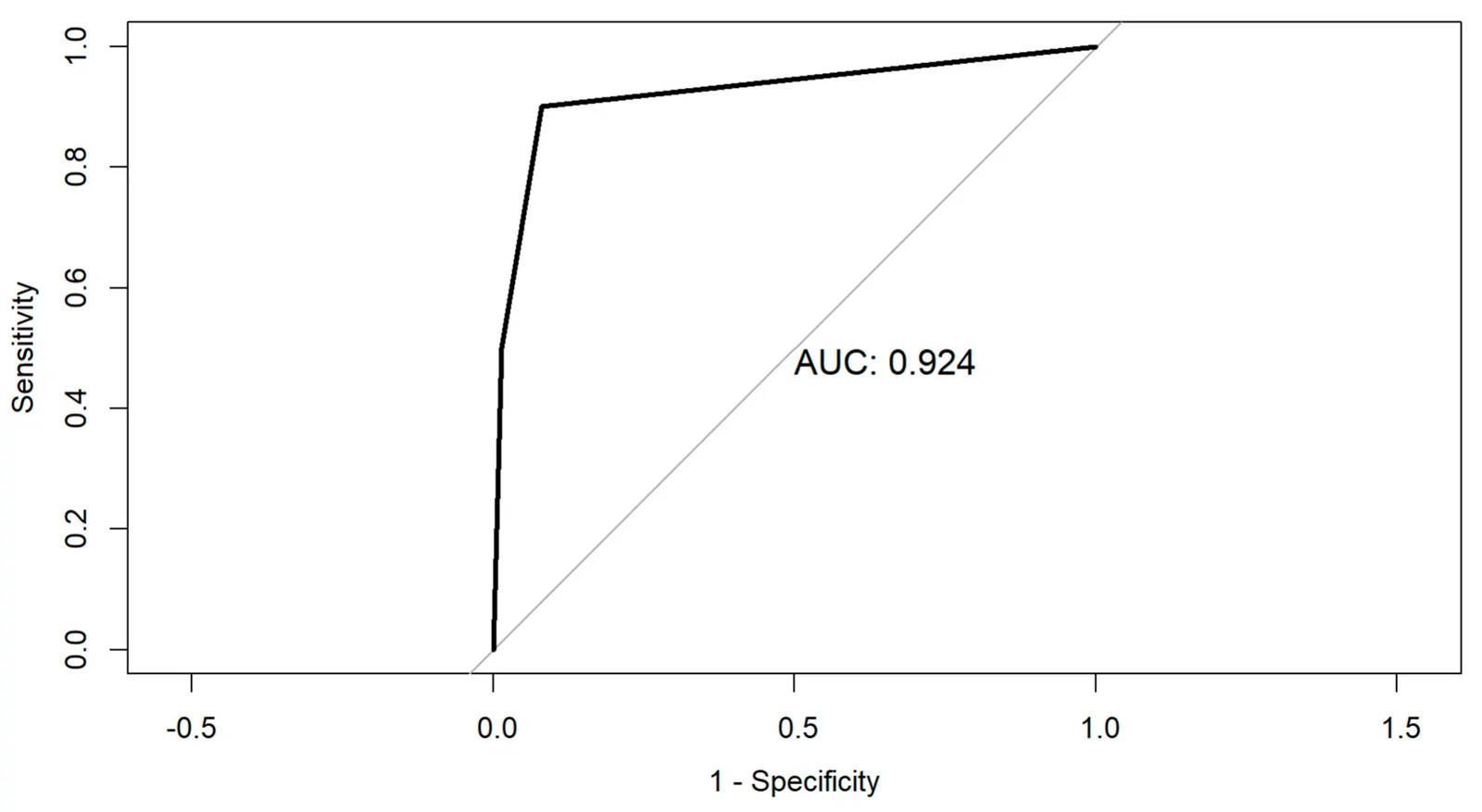
Receiver operating characteristic curve for the composite morphological score in distinguishing children with celiac disease from healthy controls. The score ranged from 0 to 2 according to the presence of PPHA and neutrophil dysplasia. At the optimal cutoff of ≥1, the AUC was 0.924, sensitivity was 90.0%, and specificity was 91.9%.

The diagnostic performance of the composite peripheral blood smear score was evaluated using ROC curve analysis (Figure 3). This score ranged from 0 to 2 according to the number of morphological abnormalities present: neither PPHA nor neutrophil dysplasia (score 0), either abnormality alone (score 1), or both abnormalities (score 2). The composite score demonstrated excellent discrimination between children with celiac disease and healthy controls, with an AUC of 0.924. At the optimal cutoff of ≥1, corresponding to the presence of at least one of the two morphological abnormalities, sensitivity was 90.0% and specificity was 91.9%. Thus, the ROC analysis reflects the combined discriminatory contribution of PPHA and neutrophil dysplasia rather than the performance of either abnormality alone.

## 4. Discussion

The present study demonstrates that children with celiac disease exhibit a remarkably high prevalence of pseudo-Pelger–Huët anomaly (PPHA) and neutrophil dysplasia compared with healthy controls. While conventional hematological and biochemical abnormalities were relatively limited, profound differences were observed in neutrophil morphology. PPHA was identified in 87.1% of children with celiac disease compared with only 8.1% of controls, whereas neutrophil dysplasia was present in 52.9% and 1.4% of subjects, respectively. Furthermore, these associations remained highly significant after adjustment for age and sex. These findings suggest that peripheral blood smear abnormalities may represent an underrecognized hematological manifestation of pediatric celiac disease.

Growth impairment remains one of the most common extraintestinal manifestations of celiac disease in childhood. In the present study, underweight status and short stature were significantly more frequent among children with celiac disease than among healthy controls. These findings are consistent with previous pediatric studies demonstrating that impaired growth continues to be an important clinical feature despite increasing recognition of nonclassical disease presentations. Çatal et al. [12] reported that growth restriction and nutritional impairment were common findings at diagnosis in children with celiac disease, reflecting the systemic consequences of chronic intestinal inflammation and malabsorption. Similarly, previous pediatric cohorts have shown that disturbances in linear growth may occur even in the absence of severe gastrointestinal symptoms, emphasizing the importance of careful anthropometric assessment in affected children.

In contrast to the marked differences observed in growth parameters, most routine hematological and biochemical variables were broadly comparable between study groups. Hemoglobin concentration, hematocrit, mean corpuscular volume, vitamin B12, folate, and vitamin D levels did not differ significantly between children with celiac disease and controls. Historically, anemia, iron deficiency, folate deficiency, and vitamin B12 deficiency have been regarded as major hematological manifestations of celiac disease [14]. Several studies have reported substantially increased frequencies of anemia and micronutrient deficiencies at diagnosis, particularly among untreated patients and those with severe mucosal damage [12,28,29]. Compared with these reports, the relatively preserved hematological profile observed in our cohort may reflect earlier diagnosis, improved nutritional monitoring, or effective implementation of a gluten-free diet in a substantial proportion of patients.

Despite a few differences in most hematological indices, platelet counts were significantly higher in children with celiac disease. Thrombocytosis has previously been described in celiac disease and is generally considered a reactive phenomenon associated with chronic inflammation, iron deficiency, or functional hyposplenism [14]. Most hematological indices and nutrient levels did not differ significantly between groups, whereas platelet count and ferritin levels were significantly higher in children with celiac disease. Previous studies have similarly reported increased rates of thrombocytosis in celiac disease [30,31]. Elevated ferritin concentrations in our cohort may reflect low-grade inflammatory activity. Furthermore, inflammation may persist despite reported adherence to a gluten-free diet, and even minimal gluten exposure may sustain immune activation without necessarily producing overt serological positivity [32,33]. Taken together, these findings indicate that conventional laboratory parameters alone may underestimate the extent of hematological involvement in pediatric celiac disease.

The most striking finding of the present study was the extraordinarily high prevalence of PPHA among children with celiac disease. Congenital Pseudo-Pelger–Huët anomaly is a rare autosomal dominant condition caused by mutations in the lamin B receptor (LBR) gene and is characterized by hyposegmented neutrophil nuclei with a largely preserved cellular function [34,35]. In contrast, PPHA is an acquired morphological abnormality associated with myelodysplastic syndromes, acute myeloid leukemia, severe infections, nutritional deficiencies, autoimmune disorders, and certain medications [24,36]. However, PPHA is not specific to clonal hematological disease. Wang et al. [24] demonstrated that it may also occur in reactive settings, while Ayan et al. [22] emphasized its association with inflammatory, infectious, and drug-related processes. The present study extends these observations by demonstrating a strong association between celiac disease and PPHA in a pediatric population.

PPHA positivity was also observed in 8.1% of controls. Although this frequency was markedly lower than the 87.1% observed in children with celiac disease, it is higher than might be expected in an otherwise healthy pediatric population. The operational ≥5% threshold may have captured low-level or transient hyposegmented forms that would not necessarily indicate persistent PPHA. In addition, the controls were selected from children attending a pediatric hematology outpatient clinic and may not fully represent a population-based healthy cohort. Active infection and known hematological disease were exclusion criteria, and smear evaluation was performed with the examiner blinded to group allocation. Nevertheless, the absence of serial smear assessment prevents us from determining whether these findings were transient or persistent. Future studies should validate the selected threshold in population-based pediatric controls and include repeated assessments. To our knowledge, this is also the first study to systematically evaluate peripheral blood smear morphology in pediatric celiac disease. While previous investigations have primarily focused on complete blood count parameters, anemia, and micronutrient deficiencies [11,12,13,37], detailed morphological assessment of neutrophils has not been reported. These findings therefore expand the recognized hematological spectrum of celiac disease and support previous observations that chronic inflammatory conditions may be associated with acquired pseudo-Pelger–Huët anomaly and dysplastic granulocytic changes [32,33]. Dysplasia encompasses a spectrum of morphological abnormalities and maturation defects arising during hematopoiesis and is most commonly discussed in the context of myelodysplastic syndromes; however, similar morphological changes may also occur in nonclonal inflammatory conditions [38].

Neutrophil dysplasia was also substantially more common among children with celiac disease. Dysplastic neutrophil morphology is frequently discussed in the context of myelodysplastic syndromes; however, dysgranulopoiesis may also occur in nonclonal disorders characterized by chronic immune activation. The coexistence of PPHA and dysplastic changes in approximately half of the celiac cohort suggests a broader disturbance of neutrophil maturation rather than an isolated nuclear segmentation abnormality. Importantly, these morphological findings occurred despite largely preserved blood counts and in the absence of clinical evidence suggesting an underlying bone marrow disorder. This observation supports the hypothesis that chronic inflammatory and immune-mediated mechanisms may influence granulopoiesis in celiac disease.

Several mechanisms may explain these findings. Prolonged or inadequately controlled celiac disease is characterized by persistent activation of innate and adaptive immune pathways, resulting in sustained production of inflammatory cytokines and systemic immune activation. This chronic inflammatory milieu may directly affect hematopoietic stem and progenitor cells in the bone marrow, inducing hematopoietic stress that disrupts normal myeloid differentiation and neutrophil maturation [39,40]. Such alterations have been described as inflammatory or emergency myelopoiesis and may promote the emergence of morphologically abnormal granulocytes, including hyposegmented neutrophils and dysplastic forms. The coexistence of pseudo-Pelger–Huët anomaly and neutrophil dysplasia in our cohort is therefore biologically plausible and may reflect systemic consequences of chronic immune-mediated inflammation extending beyond the intestinal mucosa. Although the present study was not designed to directly investigate mechanistic pathways, these observations support the hypothesis that celiac disease may influence granulopoiesis through immune-mediated effects on bone marrow function.

Nutritional deficiency represents another potential explanation for dysplastic hematological changes. Vitamin B12 and folate deficiencies are well-recognized causes of dysgranulopoiesis and may produce morphological abnormalities that mimic myelodysplastic syndromes. However, several observations argue against micronutrient deficiency as the primary driver of our findings. First, vitamin B12 and folate concentrations did not differ significantly between groups. Second, peripheral blood smear abnormalities were not associated with vitamin D deficiency, celiac antibodies, adherence to a gluten-free diet, or comorbid conditions in the celiac cohort. These findings suggest that the observed neutrophil abnormalities may reflect disease-related immune dysregulation rather than isolated nutritional deficiency.

Although a strict gluten-free diet remains the cornerstone of celiac disease management and typically results in improvement of most hematological manifestations, previous studies have shown that certain abnormalities may persist despite long-term treatment. For example, unresolved anemia has been reported in a small proportion of pediatric patients even after 8–10 years of follow-up [40]. In our cohort, although the majority of patients reported adherence to a gluten-free diet, 57.1% remained positive for celiac-related autoantibodies. This relatively high rate of seropositivity may indicate ongoing immune activation in at least a subset of patients and could partially explain the persistence of neutrophil morphological abnormalities despite otherwise preserved hematological indices.

From a clinical perspective, the diagnostic performance of peripheral blood smear abnormalities was notable. ROC analysis demonstrated excellent discrimination between children with celiac disease and controls, with an area under the curve of 0.924, sensitivity of 90.0%, and specificity of 91.9%. Although PPHA and neutrophil dysplasia cannot be considered diagnostic markers of celiac disease, their presence may increase clinical suspicion and prompt further investigation in children presenting with unexplained growth impairment or other extraintestinal manifestations. Because peripheral blood smear examination is inexpensive, readily available, and routinely performed in clinical practice, recognition of these morphological abnormalities may have practical value as an adjunctive hematological clue.

Several limitations should be acknowledged. First, the retrospective, single-center, cross-sectional design and relatively modest sample size limit the generalizability of the findings and preclude conclusions regarding causality, persistence, or reversibility. Second, although the smear examiner was blinded to group allocation, all morphological assessments were performed by a single pediatric hematologist; therefore, interobserver reliability could not be evaluated. Third, the PPHA threshold was an operational definition used in this study rather than a universally validated diagnostic cut-off and requires validation in independent pediatric populations. In addition, dysplasia and the combined outcome were observed in only one control participant, resulting in sparse data and potentially unstable estimates. Although Firth’s penalized logistic regression was used to reduce sparse-data bias, the resulting associations should be interpreted cautiously. The clinical subgroup analyses were also based on small numbers and should be considered exploratory. Furthermore, inflammatory cytokines, oxidative stress markers, and nutritional factors beyond routinely measured micronutrients were unavailable, precluding direct investigation of the underlying mechanisms. Bone marrow examination and molecular analyses were not performed; therefore, the relationship between peripheral smear abnormalities and alterations in granulopoiesis could not be evaluated directly. Future studies incorporating molecular characterization and, where clinically justified, bone marrow assessment may provide further mechanistic insight [35,41]. Finally, longitudinal follow-up data were unavailable, preventing assessment of whether PPHA and neutrophil dysplasia change following long-term adherence to a gluten-free diet. Prospective studies are warranted to determine the persistence, reversibility, and long-term clinical significance of these abnormalities [42,43].

Future research should employ a prospective, multicenter, longitudinal framework involving children with newly diagnosed treatment-naïve celiac disease and age- and sex-matched healthy controls. Peripheral blood smears should be evaluated at diagnosis and at predefined intervals after initiation of a gluten-free diet, such as 3, 6, and 12 months. Morphological assessment should follow standardized criteria and be performed independently by at least two hematologists blinded to clinical and laboratory data, with interobserver agreement formally quantified. Serial smear findings should be evaluated alongside complete blood counts, celiac serology, objective measures of dietary adherence, anthropometric recovery, iron status, vitamin B12, folate, and markers of systemic inflammation. Where feasible, assessment of inflammatory mediators implicated in celiac disease and granulopoiesis, including IL-15 and interferon-γ, may help clarify the underlying mechanisms. The primary outcomes should be the prevalence and longitudinal reversibility of PPHA and neutrophil dysplasia, whereas secondary outcomes should include their associations with disease activity, nutritional status, mucosal recovery, and treatment response. A priori sample-size calculation and external validation in an independent cohort would also be required before these morphological abnormalities could be considered clinically useful biomarkers.

## 5. Conclusions

This study demonstrates a remarkably high prevalence of pseudo-Pelger–Huët anomaly (PPHA) and neutrophil dysplasia in children with celiac disease compared with healthy controls. While conventional hematological and biochemical parameters were largely similar between groups, examination of the peripheral blood smear revealed profound differences in neutrophil morphology, suggesting that hematological involvement in celiac disease may extend beyond abnormalities detected by routine laboratory testing. Importantly, these morphological changes were not associated with vitamin D deficiency, celiac antibodies, adherence to a gluten-free diet, or comorbid conditions, suggesting they may reflect more persistent alterations in granulopoiesis rather than transient disease activity. To our knowledge, this is the first study to evaluate peripheral blood smear morphology systematically and document the high prevalence of PPHA and neutrophil dysplasia in pediatric celiac disease. The findings support the concept that chronic immune activation and inflammation in celiac disease may influence neutrophil maturation and contribute to dysplastic morphological changes in the peripheral blood. The present findings do not establish a diagnostic role for these abnormalities or demonstrate that their recognition prevents misdiagnosis of myelodysplastic syndromes. Rather, they indicate that PPHA-like and dysplastic neutrophil features warrant further investigation in pediatric celiac disease. Their clinical relevance can only be determined through prospective studies incorporating standardized morphological criteria, multiple blinded observers, appropriate control populations, and longitudinal follow-up.

## Figures and Tables

**Table 1:** Demographic and anthropometric characteristics of the study groups.

Characteristic	Celiac Disease (n = 70)	Controls (n = 74)	*p* Value
Age, years (mean ± SD)	9.79 ± 4.18	8.69 ± 4.95	0.142
Female sex, %	57.1	56.8	1.000
Underweight, % *	15.7	2.7	0.008
Short stature, %	21.4	1.4	<0.001

* Note: Underweight and short stature were defined as weight-for-age and height-for-age < −2 SD, respectively. SD: Standard deviation.

**Table 2:** Hematological and biochemical parameters in celiac disease and controls.

Parameter	Celiac Disease (n = 70)	Controls (n = 74)	*p* Value
WBC,/µL	6881.9 (6488.1–7275.7)	7104.2 (6636.9–7571.6)	0.472
RBC, ×10^6^/µL	4.5 (4.4–4.7)	4.4 (4.3–4.5)	0.023
Hemoglobin, g/dL	12.8 (12.4–13.1)	12.6 (12.4–12.9)	0.573
Hematocrit, %	39.0 (37.5–40.4)	38.4 (37.2–39.6)	0.562
MCV, fL	79.0 (77.1–80.8)	80.8 (79.6–82)	0.080
RDW, %	13.8 (13.5–14.1)	13.2 (13.1–13.4)	0.001
Platelets, /µL	334,851.4 (313,660.2–356,042.5)	305,442.9 (287,185.2–323,700.5)	0.039
Vitamin B12, pg/mL	386.7 (366.3–407.2)	406.3 (374.2–438.5)	0.313
Folate, ng/mL	11.6 (10.8–12.4)	12.0 (11.3–12.6)	0.479
Ferritin, ng/mL	30.1 (17.3–42.9)	28.6 (25.1–32)	0.008
Vitamin D, ng/mL	32.2 (30.6–33.8)	31.0 (29.3–32.7)	0.244

Note: Values are expressed as mean (95% confidence interval). Abbreviations: WBC, white blood cell; RBC, red blood cell; MCV, mean corpuscular volume; RDW, red cell distribution width.

**Table 3:** Main peripheral blood smear findings.

Finding	Celiac Disease (n = 70)	Controls (n = 74)	*p* Value
Pelger–Huët anomaly, %	87.1	8.1	<0.001
Dysplasia present, %	52.9	1.4	<0.001
PHA + dysplasia, %	50.0	1.4	<0.001

Note: Peripheral blood smears were evaluated by light microscopy during a 100-cell differential count. PHA: Pelger–Huët anomaly.

**Table 4:** Multivariable Logistic Regression Analysis of Peripheral Blood Smear Abnormalities.

Outcome	Adjusted OR * (95% CI)	*p*-Value
Pseudo-Pelger–Huët anomaly	81.19 (28.65–278.45)	<0.001
Neutrophil dysplasia	93.85 (18.46–1724.96)	<0.001
Pseudo-Pelger–Huët anomaly + dysplasia	79.89 (15.86–1461.89)	<0.001

* Adjusted for age and sex.

**Table 5:** Associations Between Peripheral Blood Smear Abnormalities and Clinical Variables in Children with Celiac Disease.

Clinical Variable	Category	Total, n	PPHA, n/N (%)	*p* Value	Neutrophil Dysplasia, n/N (%)	*p* Value
Celiac antibody status	Positive	40	36/40 (90.0)	0.483	18/40 (45.0)	0.152
	Negative	30	25/30 (83.3)		19/30 (63.3)	
Gluten-free diet adherence	Adherent	51	45/51 (88.2)	0.696	27/51 (52.9)	1.000
	Non-adherent	19	16/19 (84.2)		10/19 (52.6)	
Comorbid condition	Present	14	13/14 (92.9)	0.675	8/14 (57.1)	0.772
	Absent	56	48/56 (85.7)		29/56 (51.8)	
Vitamin D deficiency	Present	21	20/21 (95.2)	0.261	12/21 (57.1)	0.795
	Absent	49	41/49 (83.7)		25/49 (51.0)	

Values are presented as n/N (%). Comparisons were performed using Fisher’s exact test. Owing to the small subgroup sizes, these analyses should be considered exploratory.

## Data Availability

The data presented in this study are available on request from the corresponding author. The data are not publicly available due to privacy restrictions.

## Abbreviations

The following abbreviations are used in this manuscript:

AML	Acute myeloid leukemia
AUC	Area under the curve
CD	Celiac disease
CI	Confidence interval
GFD	Gluten-free diet
HCT	Hematocrit
HGB	Hemoglobin
LBR	Lamin B receptor
MCV	Mean corpuscular volume
MDSs	Myelodysplastic syndromes
OR	Odds ratio
PLT	Platelet
PPHA	Pseudo-Pelger–Huët anomaly
RBC	Red blood cell
RDW	Red cell distribution width
ROC	Receiver operating characteristic
SD	Standard deviation
WBC	White blood cell

## References

[1] Bingham S.M., Bates M.D. (2020). Pediatric celiac disease: A review for non-gastroenterologists. Curr. Probl. Pediatr. Adolesc. Health Care.

[2] Husby S., Koletzko S., Korponay-Szabó I., Kurppa K., Mearin M.L., Ribes-Koninckx C., Shamir R., Troncone R., Auricchio R., Castillejo G. (2020). European Society Paediatric Gastroenterology, Hepatology and Nutrition guidelines for diagnosing coeliac disease 2020. J. Pediatr. Gastroenterol. Nutr..

[3] Iversen R., Sollid L.M. (2023). The immunobiology and pathogenesis of celiac disease. Annu. Rev. Pathol..

[4] Rubio-Tapia A., Hill I.D., Semrad C., Kelly C.P., Greer K.B., Limketkai B.N., Lebwohl B. (2023). American College of Gastroenterology guidelines update: Diagnosis and management of celiac disease. Am. J. Gastroenterol..

[5] Dunne M.R., Byrne G., Chirdo F.G., Feighery C. (2020). Coeliac disease pathogenesis: The uncertainties of a well-known immune-mediated disorder. Front. Immunol..

[6] Singh P., Arora A., Strand T.A., Leffler D.A., Catassi C., Green P.H.R., Kelly C.P., Ahuja V., Makharia G.K. (2018). Global prevalence of celiac disease: Systematic review and meta-analysis. Clin. Gastroenterol. Hepatol..

[7] Almallouhi E., King K.S., Patel B., Wi C., Juhn Y.J., Murray J.A., Absah I. (2017). Increasing incidence and altered presentation in a population-based study of pediatric celiac disease in North America. J. Pediatr. Gastroenterol. Nutr..

[8] Durazzo M., Ferro A., Brascugli I., Mattivi S., Fagoonee S., Pellicano R. (2022). Extra-intestinal manifestations of celiac disease: What should we know in 2022?. J. Clin. Med..

[9] Mearin M.L., Agardh D., Antunes H., Al-Toma A., Auricchio R., Castillejo G., Cellier C., Dolinsek J., Hadjivassiliou M., Kurppa K. (2022). ESPGHAN position paper on management and follow-up of children and adolescents with celiac disease. J. Pediatr. Gastroenterol. Nutr..

[10] Bolia R., Thapar N. (2024). Celiac disease in children: A 2023 update. Indian J. Pediatr..

[11] Balaban D.V., Popp A., Ionita-Radu F., Jinga M. (2019). Hematologic manifestations in celiac disease—A practical review. Medicina.

[12] Çatal F., Topal E., Ermiştekin H., Yildirim Acar N., Sinanoğlu M.S., Karabiber H., Selimoğlu M.A. (2015). The hematologic manifestations of pediatric celiac disease at the time of diagnosis and efficiency of gluten-free diet. Turk. J. Med. Sci..

[13] Madni B., Malhi K.A., Rehman F., Shahnawaz K., Zahoor F., Mughal B.B. (2019). Hematological manifestations of celiac disease in children. Med. Forum.

[14] Halfdanarson T.R., Litzow M.R., Murray J.A. (2007). Hematologic manifestations of celiac disease. Blood.

[15] Baydoun A., Maakaron J.E., Halawi H., Abou Rahal J., Taher A.T. (2012). Hematological manifestations of celiac disease. Scand. J. Gastroenterol..

[16] Martín-Masot R., Nestares M.T., Diaz-Castro J., López-Aliaga I., Alférez M.J.M., Moreno-Fernandez J., Maldonado J. (2019). Multifactorial etiology of anemia in celiac disease and effect of gluten-free diet: A comprehensive review. Nutrients.

[17] Kreutz J.M., Adriaanse M.P., van der Ploeg E.M., Vreugdenhil A.C. (2020). Narrative review: Nutrient deficiencies in adults and children with treated and untreated celiac disease. Nutrients.

[18] Manley H.R., Keightley M.C., Lieschke G.J. (2018). The neutrophil nucleus: An important influence on neutrophil migration and function. Front. Immunol..

[19] Invernizzi R. (2021). Images from the Haematologica Atlas of Hematologic Cytology: Dysgranulopoiesis. Haematologica.

[20] Shekhar R., Srinivasan V.K., Pai S. (2021). How I investigate dysgranulopoiesis. Int. J. Lab. Hematol..

[21] Colella R., Hollensead S.C. (2012). Understanding and recognizing the Pelger-Huët anomaly. Am. J. Clin. Pathol..

[22] Ayan M.S., Abdelrahman A.M.M., Khanal N., Elsallabi O.S., Birch N.C. (2015). Case of acquired or pseudo-Pelger-Huët anomaly. Oxf. Med. Case Rep..

[23] Hoffmann K., Dreger C.K., Olins A.L., Olins D.E., Shultz L.D., Lucke B., Karl H., Kaps R., Müller D., Vayá A. (2002). Mutations in the gene encoding the lamin B receptor produce an altered nuclear morphology in granulocytes (Pelger–Huët anomaly). Nat. Genet..

[24] Wang E., Boswell E., Siddiqi I., Lu C.M., Sebastian S., Rehder C., Huang Q. (2011). Pseudo–Pelger-Huët anomaly induced by medications: A clinicopathologic study in comparison with myelodysplastic syndrome-related pseudo–Pelger-Huët anomaly. Am. J. Clin. Pathol..

[25] Dahele A., Ghosh S. (2001). Vitamin B12 deficiency in untreated celiac disease. Am. J. Gastroenterol..

[26] Abadie V., Jabri B. (2014). IL-15: A central regulator of celiac disease immunopathology. Immunol. Rev..

[27] Watson A., Kolinski J., Suchi M., Elkadri A. (2021). Bone marrow suppression associated with celiac disease in a 4-year-old boy. ACG Case Rep. J..

[28] Rajalahti T., Repo M., Kivelä L., Huhtala H., Mäki M., Kaukinen K., Kurppa K. (2017). Anemia in pediatric celiac disease: Association with clinical and histological features and response to gluten-free diet. J. Pediatr. Gastroenterol. Nutr..

[29] Repo M., Lindfors K., Mäki M., Huhtala H., Laurila K., Lähdeaho M.L., Kurppa K. (2017). Anemia and iron deficiency in children with potential celiac disease. J. Pediatr. Gastroenterol. Nutr..

[30] Lebwohl B., Sanders D.S., Green P.H.R. (2018). Coeliac disease. Lancet.

[31] Pantic N., Pantic I., Jevtic D., Mogulla V., Oluic S., Durdevic M., Nordin T., Jecmenica M., Milovanovic T., Gavrancic T. (2022). Celiac disease and thrombotic events: Systematic review of published cases. Nutrients.

[32] Barone M.V., Auricchio R., Nanayakkara M., Greco L., Troncone R., Auricchio S. (2022). Pivotal role of inflammation in celiac disease. Int. J. Mol. Sci..

[33] Kotze L.M.D.S., Kotze L.R., Moreno I., Nisihara R. (2018). Immune-mediated diseases in patients with celiac disease and their relatives: A comparative study of age and sex. Arq. Gastroenterol..

[34] Speeckaert M.M., Verhelst C., Koch A., Speeckaert R., Lacquet F. (2009). Pelger-Huët anomaly: A critical review of the literature. Acta Haematol..

[35] Konishi T., Muto H., Sakuma R., Miyawaki S., Ohashi K., Doki N., Tomiyama J. (2019). Familial case of hereditary Pelger-Huët anomaly. Int. J. Hematol..

[36] Savaşan S., Buck S., Gadgeel M., Poulik J. (2021). Persistent pseudo-Pelger-Huët anomaly. Ann. Hematol..

[37] Özdemir Z.C., Düzenli Kar Y., Bektaş P.C., Eren M., Bilgin M., Bör Ö. (2021). Hematological parameters and leukocyte formulas in predicting celiac disease in children with iron deficiency anemia. J. Acad. Res. Med..

[38] Hamsho S., Hariri M.A., Sleiay B., Alhussen A.H.D., Sleiay M. (2024). Is there any association between celiac disease, myelodysplastic syndrome and primary sclerosing cholangitis? A rare case report. JGH Open.

[39] Cain D.W., Snowden P.B., Sempowski G.D., Kelsoe G. (2011). Inflammation triggers emergency granulopoiesis through a density-dependent feedback mechanism. PLoS ONE.

[40] Paudel S., Ghimire L., Jin L., Jeansonne D., Jeyaseelan S. (2022). Regulation of emergency granulopoiesis during infection. Front. Immunol..

[41] Patel S.S. (2021). Pediatric myelodysplastic syndromes. Clin. Lab. Med..

[42] Valvano M., Giansante C., Vinci A., Maurici M., Fabiani S., Stefanelli G., Cesaro N., Viscido A., Caloisi C., Latella G. (2025). Persistence of anemia in patients with celiac disease despite a gluten-free diet: A retrospective study. BMC Gastroenterol..

[43] Agin M., Kayar Y., Dertli R., Konur S., Surmeli N., Ozkahraman A. (2020). The effect of gluten-free diet on mean platelet volume, neutrophil and neutrophil/lymphocyte ratio in children with celiac disease. Ann. Med. Res..

